# Supplementary material for: Reduction of elevated proton leak rejuvenates mitochondria in the aged cardiomyocyte
Source: eLife. 2020 Dec 15;9:e60827. doi: 10.7554/eLife.60827 (PMC7738186; doi:10.7554/eLife.60827)
Supplement: Transparent reporting form [file elife-60827-transrepform.docx]

***eLife’s* transparent reporting form**

We encourage authors to provide detailed information *within their submission* to facilitate the interpretation and replication of experiments. Authors can upload supporting documentation to indicate the use of appropriate reporting guidelines for health-related research (see [EQUATOR Network](http://www.equator-network.org/%20)), life science research (see the [BioSharing Information Resource](https://biosharing.org/)), or the [ARRIVE guidelines](http://www.plosbiology.org/article/info:doi/10.1371/journal.pbio.1000412) for reporting work involving animal research. Where applicable, authors should refer to any relevant reporting standards documents in this form.

If you have any questions, please consult our Journal Policies and/or contact us: [editorial@elifesciences.org](mailto:editorial@elifesciences.org).

**Sample-size estimation**

- You should state whether an appropriate sample size was computed when the study was being designed
- You should state the statistical method of sample size computation and any required assumptions
- If no explicit power analysis was used, you should describe how you decided what sample (replicate) size (number) to use

Please outline where this information can be found within the submission (e.g., sections or figure legends), or explain why this information doesn’t apply to your submission:

Sample size was determined based on results of similar studies in this field, and most importantly, our experience with the techniques performed, the variance of the assays and the magnitude of observed differences between ages and treatment conditions. These determined the sample sizes required to achieve statistical significance. Sample numbers (N) and the statistical tests used are given in every figure legend.

**Replicates**

- You should report how often each experiment was performed
- You should include a definition of biological versus technical replication
- The data obtained should be provided and sufficient information should be provided to indicate the number of independent biological and/or technical replicates
- If you encountered any outliers, you should describe how these were handled
- Criteria for exclusion/inclusion of data should be clearly stated
- High-throughput sequence data should be uploaded before submission, with a private link for reviewers provided (these are available from both GEO and ArrayExpress)

Please outline where this information can be found within the submission (e.g., sections or figure legends), or explain why this information doesn’t apply to your submission:

All the information is in the methods or figure legends. This is replicated below:

Figure 1A: the average trace of Seahorse Assay from Young 7 mice; Old 14 mice; Old+100nM SS31 10 mice. Figure 1B, C, D: Young N=7; Old N=14; Old+100nM SS31 N=10; Old+BKA N=5; Old+CAT N=5. There are 1-5 wells (technical replicates) per biological replicate. The average of the technical replicates is used as the value of the biological replicate.

Figure 2A: The representative image of pH stress. Figure 2B, C, E, F: Each biological replicate is the average of about 10 cells from 1 rat. Figure 2B: The average trace of pH stress data from Young N=4; Old N=19; Old+SS31 N=8. Figure 2C: The cpYFP 488/405 ratio at pH 5.3 from Young N=5; Old N=19; Old+SS31 N=8. Figure 2D: The slope was determined by the formula from Excel software. The slope of cpYFP decrease at pH 6.9 from Old N=10; Old+SS31 N=4. Figure 2E: SS31 time dependent effect determined at pH 5.3 from Young N=4; Old N=4; Old+SS31 at 1min N=4; Old+SS31 at 4-5min N=3; Old+SS31 at 7-10min N=4; Old+SS3 at 20-40 min N=4. Figure 2F: SS-31 dose effect determined at pH 5.3 from Young N=5; Old N=14; SS-31 1nM N=4, SS-31 10nM N=3; SS-31 100nM N=4, SS-31 1µM N=4, SS-31 10µM N=8.

Figure 3A:cpYFP 488/405 ratio at pH 5.3 from Young N=5; Old N=19; Old+BKA N=6; Old+CAT N=7; Old+Genipin N=4; Old+OA N=4. Figure 3B: The slope of cpYFP 488/405 ratio after pH 6.9. Old N=10; Old+BKA N=6; Old+CAT N=5; Old+Genipin N=4; Old+OA N=4.

Figure 4A,B,C: singled cells and traces shown for illustrative purposes. Figure 4D: The mitochondrial flash: the value is derived from the number of flashes observed during 100s scanning in 1000 square micrometer of cell area. Young, 28 cells from 5 rats. Old, 87 cells from 14 rats; Old+SS31, 26 cells from 3 rats; Old+BKA, 40 cells from 4 rats; 37 cells from 3 rats. Figure 4E: The mitochondrial membrane potential evaluated by JC-1. Young, 84 cells from 3 mice; Old, 218 cells from 4 mice; Old+SS31, 217 cells from 3 mice; Figure 4F: Mitochondrial ROS evaluation. Young, 40 cells from 3 mice; Old, 61 cells from 5 mice; Old+SS31, 84 cells from 5 mice.

Figure5A: A representative image show the photon induced MPTP opening. The time to mPTP opening value is based on a linear scall over time of TMRM fluorescent lasting. Figure 5B: The average time to opening is derived from: Young, 582 mitochondria from 25 cells isolated from 4 mice; Old, 591 mitochondria from 26 cells isolated from 4 mice; Old+SS31, 418 mitochondria from 19 cells isolated from 3 mice; Old+BKA, 658 mitochondria from 32 cells isolated from 3 mice; Old+BKA, 646 mitochondria from 31 cells isolated from 3 mice.

Figure 6 A, C and D. Representative blots or native gels are shown, with each lane derived from an independent animal heart preparation. Figure 6B and 6E. Each dot is the ratio of density of indicated bands, each dot derived from analysis of tissue from an individual animal, N=6 animals in figure 6B and N=8 in figure 6E. In Figure 6B values are shown relative to the density of the ANT1 band obtained with 10μM Biotin-SS-31 (without competition with other agents); the10μM Biotin-SS-31 band is thus always 1.0.

Figure1 S1: There are 2-5 wells (technical replicates) per biological replicate. The average of the technical replicates is used as one value per biological replicate. N=5 in each group.

Figure1 S2: There are 1-5 wells (technical replicates) per biological replicate. The average of the technical replicates is used as one value per biological replicate. Young N=7; Old N=14; Old+1nM SS31 N=8; Old+10nM SS31 N=8; Old+100nM SS31 N=10; Old+1µM SS31 N=7; Old+10 µM SS31 N=6.

Figure2 S1: Each value is the average of 16 cells in each pH level.

Figure2 S2: The cpYFP 488/405 ratio at pH 6.9 from Young N=5; Old N=18; Old+SS31 N=8.

Figure2 S3 and Figure 3 S2: This are result of analysis of a single biological sample, used for illustrative purposes only.

Figure3 S1: Each lane and each data point is from an independent mouse, N=6 mice in each group.

Figure4 S1: the value is derived from the number of flashes observed during 100s scanning in 1000 square micrometer of mitochondrial area.in each scan region. Young, N= 18 from 3 mice; Old, N=16 from 4 mice

Figure6 S1: This is an image of a representative experiment.

**Statistical reporting**

- Statistical analysis methods should be described and justified
- Raw data should be presented in figures whenever informative to do so (typically when N per group is less than 10)
- For each experiment, you should identify the statistical tests used, exact values of N, definitions of center, methods of multiple test correction, and dispersion and precision measures (e.g., mean, median, SD, SEM, confidence intervals; and, for the major substantive results, a measure of effect size (e.g., Pearson's r, Cohen's d)
- Report exact p-values wherever possible alongside the summary statistics and 95% confidence intervals. These should be reported for all key questions and not only when the p-value is less than 0.05.

Please outline where this information can be found within the submission (e.g., sections or figure legends), or explain why this information doesn’t apply to your submission:

All the statistical methods and individual number of samples analyzed are indicated in the Figure legends. Individual data points were included in graphs when sample sizes were less than 10 per group. Each bar shows the Mean+/-SEM in all figures. Excel software was used to perform statistical analyses. For the multiple groups comparisons one-way ANOVA was applied, followed with the appropriate post-hoc test, either Fishers Least Significant Difference (LSD) or Dunnett’s test, as indicated. Student’s t-test was used to determine the statistical significance between two group comparisons. P < 0.05 was considered statistically significant. Figure6B, 6E, S1, S6, were graphed by GraphPad Prism. Other quantitative data were graphed by excel software.

(For large datasets, or papers with a very large number of statistical tests, you may upload a single table file with tests, Ns, etc., with reference to sections in the manuscript.)

**Group allocation**

- Indicate how samples were allocated into experimental groups (in the case of clinical studies, please specify allocation to treatment method); if randomization was used, please also state if restricted randomization was applied
- Indicate if masking was used during group allocation, data collection and/or data analysis

Please outline where this information can be found within the submission (e.g., sections or figure legends), or explain why this information doesn’t apply to your submission:

This is not applicable, as mice were obtained from the NIA rodent resource, randomly selected by them according to the ages shown. Animals were censored (euthanized) only if required by IACUC criteria of pain or distress due to health conditions. Cells or tissue from each animal were randomly distributed to treatment groups or conditions.

**Additional data files (“source data”)**

- We encourage you to upload relevant additional data files, such as numerical data that are represented as a graph in a figure, or as a summary table
- Where provided, these should be in the most useful format, and they can be uploaded as “Source data” files linked to a main figure or table
- Include model definition files including the full list of parameters used
- Include code used for data analysis (e.g., R, MatLab)
- Avoid stating that data files are “available upon request”

Please indicate the figures or tables for which source data files have been provided:

All the original data and statistic figures are updated in Dryad and accessible in the following google drive:

<https://drive.google.com/drive/u/0/folders/111fHzdpiB53emmarVSrAc5v6VVl933TL>

The confocal images can be viewed by Fiji Software which is also included in the google drive.
